# Synthesis, FT–IR characterization and crystal structure of aqua­(5,10,15,20-tetra­phenyl­porphyrinato-κ^4^ *N*)manganese(III) tri­fluoro­methane­sulfonate

**DOI:** 10.1107/S2056989016006630

**Published:** 2016-04-22

**Authors:** Wafa Harhouri, Chadlia Mchiri, Shabir Najmudin, Cecilia Bonifácio, Habib Nasri

**Affiliations:** a Laboratoire de Physico-chimie des Matériaux, Faculté des Sciences de Monastir, Avenue de l’Environnement, 5019 Monastir, University of Monastir, Tunisia; b Faculdade de Medicina, Veterinària, Universidade Tecnica de Lisboa, Avenida da Universidade Tecnica, 1300-477 Lisboa, Portugal; c REQUIMTE/CQFB Departamento de Quimica, Faculdade de Ciencias e Tecnologia, Universidade Nova de Lisboa, 2829-516 Caparica, Portugal

**Keywords:** crystal structure, hydrogen bonds, manganese(III) porphyrin complex

## Abstract

In the title salt, [Mn(C_44_H_28_N_4_)(H_2_O)](CF_3_SO_3_) or [Mn^III^(TPP)(H_2_O)](CF_3_SO_3_) (where TPP is the dianion of 5,10,15,20-tetra­phenyl­porphyrin), the Mn^III^ cation is chelated by the four pyrrole N atoms of the porphyrinate anion and additionally coordinated by an aqua ligand in an apical site, completing the distorted square-pyramidal coordination environment. The average Mn—N(pyrrole) bond length is 1.998 (9) Å and the Mn—O(aqua) bond length is 2.1057 (15) Å. The central Mn^III^ ion is displaced by 0.1575 (5) Å from the N_4_C_20_ mean plane of the porphyrinate anion towards the apical aqua ligand. The porphyrinate macrocycle exhibits a moderate ruffling and strong saddle deformations. In the crystal lattice, the [Mn^III^(TPP)(H_2_O)]^+^ cation and the tri­fluoro­methane­sulfonate counter-ions are arranged in alternating planes packed along [001]. The components are linked together through O—H⋯O hydrogen bonds and much weaker C—H⋯O and C—H⋯F inter­actions. The crystal packing is further stabilized by weak C—H⋯π inter­actions involving the pyrrole and phenyl rings of the porphyrin moieties.

## Abstract 2

This porphyrinate macrocycle of the title compound exhibits a strong saddle and moderate ruffling deformations. In the crystal, the individual manganese porphyrin complex cations and the tri­fluoro­methane­sulfonate anions are arranged in alternating planes stacked along [001].

## Chemical context   

While the role of manganese porphyrins in biological processes has not been unambiguously established (Boucher *et al.*, 1972 ▸), synthetic manganese porphyrin complexes have been used extensively as models for monoxygenases enzymes (Meunier *et al.*, 1988 ▸; Groves & Nemo, 1983 ▸) or as DNA cleavage agents (Rodriguez & Bard, 1992 ▸; Bernadou *et al.*, 1989 ▸). The latter can also be considered as potential contrast enhancement agents for magnetic resonance imaging (Fawwaz *et al.*, 1990 ▸).
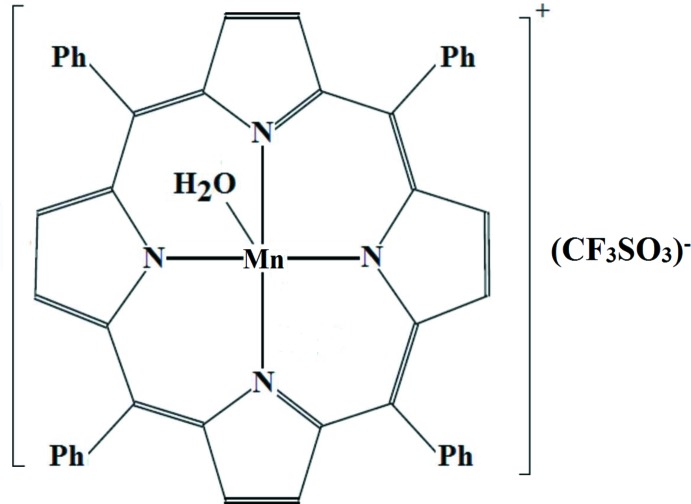


In most Mn^III^–porphyrin complexes, the metal is five-coordinate and is in its high-spin state whereby polar solvents readily can displace the coordinating anionic ligand to yield solvated complexes (Godziela *et al.*, 1986 ▸; Janson *et al.*, 1973 ▸). In our case, the reaction of chlorido-(5,10,15,20-tetra­phenyl­porphyrinato)manganese(III) with hygroscopic silver triflate let to the formation of an aqua-[5,10,15,20-tetra­phenyl­porphyrinato)]manganese(III) salt, [Mn(C_44_H_28_N_4_)(H_2_O)](CF_3_SO_3_), (I) or [Mn^III^(TPP)(H_2_O)](CF_3_SO_3_) (where TPP is the dianion of 5,10,15,20-tetra­phenyl­porphyrin). The coord­in­ation of a water mol­ecule instead of the triflate ion to Mn^III^ can be explained, as mentioned above, by the weak affinity of manganese(III) to an ionic ligand and in particular by the triflate anion which is known to be a weakly coordinating ligand.

In order to gain more insight into the structure of aqua–Mn^III^ metalloporphyrins, we report herein the synthesis, crystal structure and the spectroscopic data of compound (I).

## Structural commentary   

**Figure 1:**
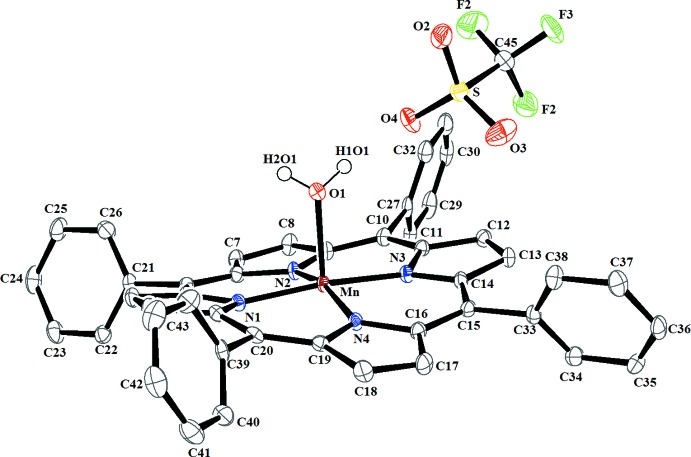
The structures of the mol­ecular entities in compound (I). Displacement ellipsoids are drawn at the 50% probability level and H atoms except those of the aqua ligand have been omitted for clarity.

**Figure 2:**
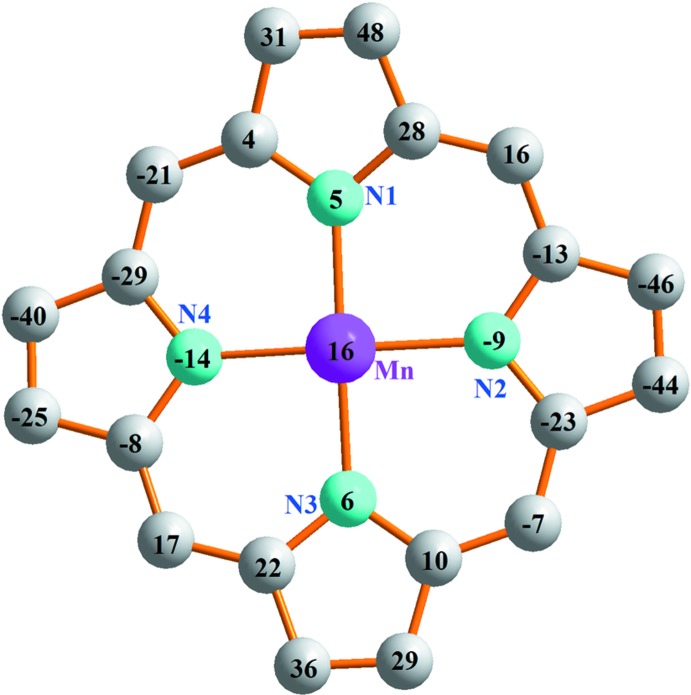
Formal diagram of the porphyrinate core illustrating the displacements of each atom from the 24-atoms core plane in units of 0.01 Å.

The central Mn^III^ cation of the complex [Mn^III^(TPP)(H_2_O)] cation exhibits a distorted square-pyramidal coordination environment (Fig. 1 ▸). The equatorial plane is formed by four nitro­gen atoms of the porphyrin ligand while the apical position is occupied by the aqua ligand. The asymmetric unit of (I) is completed by one CF_3_SO_3_^−^ counter-ion. The Mn—O(aqua) bond length of 2.1057 (15) Å is considerably shorter than those of other aqua–Mn^III^ metalloporphyrins which range from 2.166 to 2.258 Å (Dawe *et al.*, 2005 ▸; Turner, *et al.*, 1996 ▸). The average equatorial manganese–N(pyrrole) distance is 1.998 (9) Å, which is close to related [Mn^III^(Porph)(*X*)]^+^ ion complexes (Porph and *X* are a porphyrinato and a monodentate neutral ligand, respectively), *e.g.* [Mn^III^(TClPP)(py)]^+^ (TClPP is 5,10,15,20-(tetra-4-chloro­phen­yl)porphyrinato) where the average Mn—N(pyrrole) bond length is 2.007 (2) Å (Rittenberg *et al.*, 2000 ▸). In Fig. 2 ▸, the displacements of each atom in (I) from the mean plane of the 24-atom porphyrin macrocycle in units of 0.01 Å is illustrated. The Mn^III^ ion is displaced by 0.158 (5) Å from the 24-atom porphyrin mean plane (*P*_C_) which is slightly higher than in the [Mn^III^(DBHPP)(H_2_O)]^+^ (DBHPP = 5,10,15,20-(3,5-di-*t*-butyl-4-hy­droxy­phen­yl)porphyrinato) species (Mn—*P*_C_ = 0.122 Å), but smaller than in the [Mn^III^(TPP)(py)]^+^ ion complex (Mn—*P*_C_ = 0.199 Å; Dawe *et al.*, 2005 ▸). As can be seen in Fig. 2 ▸, the porphyrin core presents (i) high *saddle* distortions as seen by the displacements of the pyrrole rings alternately above and below the mean porphyrin macrocycle and (ii) a moderate *ruffling* which is indicated by the high values of the displacements of the *meso*-C atoms above and below the porphyrin mean plane (Scheidt & Lee, 1987 ▸).

## Supra­molecular features   

**Figure 3:**
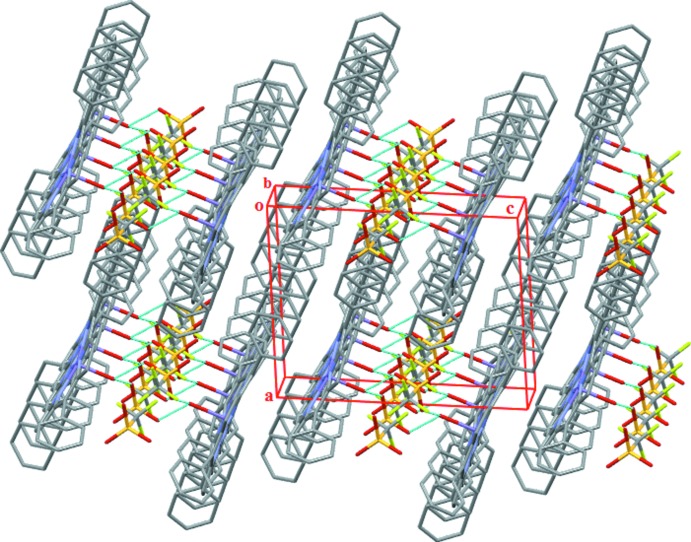
The crystal structure of the title compound in a projection approximately along [010]. H atoms have been omitted.

**Figure 4:**
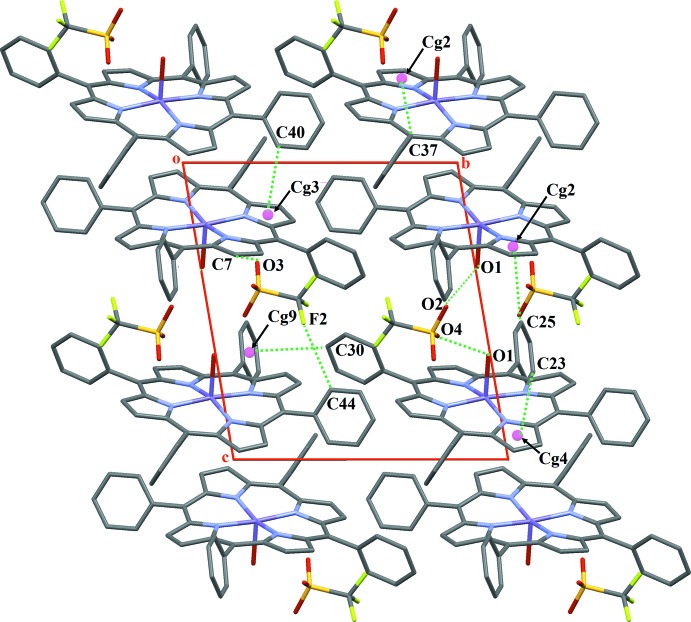
The crystal packing of (I), viewed down [100], showing the weak C—H⋯O and C—H⋯F hydrogen bonds and the C—H⋯π inter­molecular inter­actions.

In the crystal packing of (I), the manganese porphyrin complex cations and the triflate anions are arranged in alternating planes packed along [001] (Fig. 3 ▸). The distance between the C_20_N_4_Mn mean planes (porphyrin cores) of two neighbouring [Mn(TPP)H_2_O)]^+^ cation complexes is 4.677 Å. The cationic and anionic entities are linked together through two O—H⋯O hydrogen bonds of medium strength between the aqua ligand and the O atoms of the triflate anion (Table 1 ▸, Fig. 3 ▸). The crystal packing of (I) is further consolidated by weak C—H⋯O and C—H⋯F hydrogen-bonding and C—H⋯π inter­actions involving the phenyl and pyrrole rings. The values of these inter­actions range between 3.449 (2) Å and 3.676 (3) Å (Table 1 ▸, Fig. 4 ▸).

## Database survey   

A search of the Cambridge Structural Database (CSD, Version 5.31; Groom *et al.*, 2016 ▸) revealed (i) eight di­aqua–Mn^III^ metalloporphyrins, *e.g.* the [Mn^III^(TPP)(H_2_O)_2_]^+^ cation (Byrn *et al.*, 1993 ▸) and (ii) two mono-aqua-=Mn^III^ porphyrins, *e.g.* the [Mn^III^(TPP)(H_2_O)]^+^ cation (Diskin-Posner *et al.*, 1999 ▸) and the [Mn^III^(DBHPP)(H_2_O)]^+^ cation [DBHPP = 5,10,15,20-(3,5-di-*t*-butyl-4-hy­droxy­phen­yl)porphyrinato; Dawe *et al.*, 2005 ▸].

## Synthesis and crystallization   

To a solution of [Mn^III^(TPP)Cl] (100 mg, 0.142 mmol) (Cheng & Scheidt, 1996 ▸) in chloro­form (10 ml) was added an excess of one equivalent of silver triflate (100 mg, 0.389 mmol). The reaction mixture was stirred at room temperature for 12 h. Crystals of the title complex were obtained by diffusion of hexa­nes through the chloro­form solution. We assume that water was delivered from the hygroscopic silver triflate salt.

Spectroscopic analysis: UV–vis spectrum in chloro­form: λ_max_ (nm) 386, 474, 570 and 604.

## FT–IR spectroscopy   

**Figure 5:**
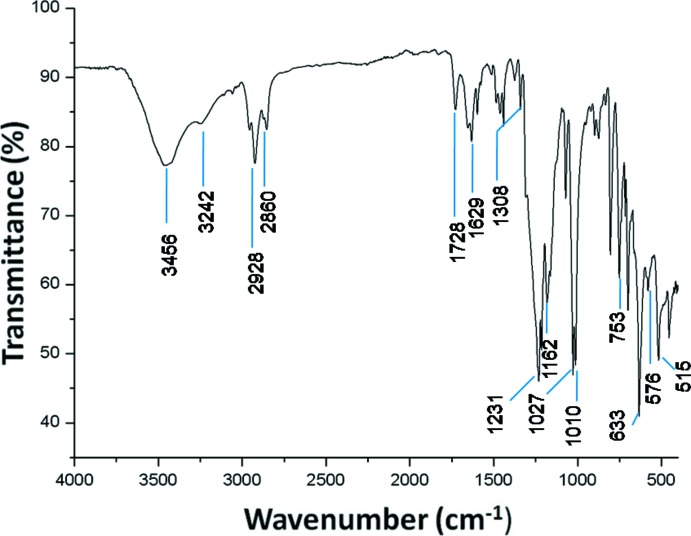
The FT–IR spectrum of (I).

The FT–IR spectrum of (I) (Fig. 5 ▸) was recorded in the 4000–400 cm^−1^ range using a PerkinElmer Spectrum Two FTIR spectrometer. The spectrum presents characteristic vibrational bands of the TPP porphyrinato moiety. The C—H stretching frequencies of the porphyrin mol­ecule are in the range 3060 to 2860 cm^−1^, the C=C and C=N stretching frequencies are assigned at 1728 cm^−1^ and 1654 cm^−1^, respectively. A strong band attributed to the bending vibration of the CCH moieties of the porphyrin core is centred around 1010 cm^−1^. The two absorption bands at 3456 cm^−1^ and 3242 cm^−1^ are attributed to the anti­symmetric and symmetric OH stretching frequencies of the aqua ligand, while the bending vibration of the same ligand is at 1629 cm^−1^. The presence of the triflate counter-ion is confirmed by the following absorption bands: a medium–strong band at 1308 cm^−1^ attributed to the asymmetric stretching frequency of the SO_3_ group, a strong band at 1231 cm^−1^ corresponding to the symmetric stretching frequency of the CF_3_ moiety, a medium–strong band at 1162 cm^−1^ attributed to ν_as_(CF_3_), a strong band at 1027 cm^−1^ corresponding to ν_s_(SO_3_), a strong band at 633 cm^−1^ attributed to the bending vibration of the SO_3_ group and a weak and a medium–strong band at 576 cm^−1^ and 515 cm^−1^ corresponding to δ_as_(CF_3_) and δ_as_(SO_3_) vibrations, respectively.

## Refinement details   

Crystal data, data collection and structure refinement details are summarized in Table 2 ▸. Carbon-bound hydrogen atoms were placed in calculated positions and refined as riding atoms with C—H = 0.93 Å with *U*_iso_(H) = 1.2*U*_eq_(C). The two hydrogen-atom positions of the aqua ligand were discernible from difference maps. However, for the final model these positions were calculated by using the *CALC-OH* program (Nardelli *et al.*, 1999 ▸) and were modelled with fixed isotropic displacement parameters.

## Supplementary Material

Crystal structure: contains datablock(s) I. DOI: 10.1107/S2056989016006630/wm5285sup1.cif

Structure factors: contains datablock(s) I. DOI: 10.1107/S2056989016006630/wm5285Isup2.hkl

CCDC reference: 1474973

Additional supporting information:  crystallographic information; 3D view; checkCIF report

## Figures and Tables

**Table 1:** Hydrogen-bond geometry (Å, °) *Cg*2, *Cg*3, *Cg*4, *Cg*7, *Cg*9 and *Cg*11 are the centroids of the N2/C6–C9, N3/C11–C14, N4/C16–C19, Mn/N2/C9–C11/N3, C21–C26 and C33–C38 rings, respectively.

*D*—H⋯*A*	*D*—H	H⋯*A*	*D*⋯*A*	*D*—H⋯*A*
O1—H1*O*1⋯O4	0.84	1.91	2.745 (2)	171
O1—H2*O*1⋯O2^i^	0.82	1.90	2.715 (2)	171
C7—H7⋯O3^ii^	0.93	2.39	3.170 (3)	141
C44—H44⋯F2^i^	0.93	2.50	3.397 (3)	162
C23—H23⋯*Cg*4^ii^	0.93	2.85	3.603 (3)	139
C25—H25⋯*Cg*2^iii^	0.93	2.89	3.650 (3)	139
C30—H30⋯*Cg*9^iv^	0.93	2.82	3.610 (3)	144
C37—H37⋯*Cg*2^v^	0.93	2.97	3.676 (3)	133
C40—H40⋯*Cg*3^vi^	0.93	2.62	3.449 (2)	148
C42—H42⋯*Cg*11^vii^	0.93	2.89	3.631 (3)	137

Symmetry codes: (i) 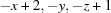; (ii) 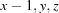; (iii) 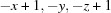; (iv) 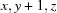; (v) 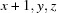; (vi) 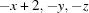; (vii) 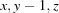.

**Table 2:** Experimental details

Crystal data	
Chemical formula	[Mn(C_44_H_28_N_4_)(H_2_O)](CF_3_O_3_S)
*M*_r_	834.76
Crystal system, space group	Triclinic, *P* 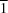
Temperature (K)	296
*a*, *b*, *c* (Å)	11.0909 (1), 12.9169 (1), 13.7931 (1)
α, β, γ (°)	78.333 (3), 81.162 (4), 74.179 (3)
*V* (Å^3^)	1851.66 (5)
*Z*	2
Radiation type	Mo *K*α
μ (mm^−1^)	0.48
Crystal size (mm)	0.48 × 0.38 × 0.16

Data collection	
Diffractometer	Bruker APEXII CCD
Absorption correction	Multi-scan (*SADABS*; Bruker, 2008 ▸)
*T*_min_, *T*_max_	0.835, 0.862
No. of measured, independent and observed [*I* > 2σ(*I*)] reflections	44659, 6753, 5533
*R*_int_	0.059
(sin θ/λ)_max_ (Å^−1^)	0.602

Refinement	
*R*[*F* ^2^ > 2σ(*F* ^2^)], *wR*(*F* ^2^), *S*	0.036, 0.086, 1.05
No. of reflections	6753
No. of parameters	523
H-atom treatment	H-atom parameters constrained
Δρ_max_, Δρ_min_ (e Å^−3^)	0.26, −0.41

Computer programs: *APEX2* and *SAINT* (Bruker, 2008 ▸), *SIR2004* (Burla *et al.*, 2005 ▸), *SHELXL2014* (Sheldrick, 2015 ▸), *ORTEPIII* (Burnett & Johnson, 1996 ▸), *ORTEP-3 for Windows* and *WinGX* (Farrugia, 2012 ▸).

## supplementary crystallographic information

### Crystal data

**Table:**

[Mn(C_44_H_28_N_4_)(H_2_O)](CF_3_O_3_S)	*Z* = 2
*M**_r_* = 834.76	*F*(000) = 856
Triclinic, *P*1	*D*_x_ = 1.497 Mg m^−^^3^
*a* = 11.0909 (1) Å	Mo *K*α radiation, λ = 0.71073 Å
*b* = 12.9169 (1) Å	Cell parameters from 9884 reflections
*c* = 13.7931 (1) Å	θ = 2.3–25.3°
α = 78.333 (3)°	µ = 0.48 mm^−^^1^
β = 81.162 (4)°	*T* = 296 K
γ = 74.179 (3)°	Plate, blue
*V* = 1851.66 (5) Å^3^	0.48 × 0.38 × 0.16 mm

### Data collection

**Table:**

Bruker APEXII CCD diffractometer	5533 reflections with *I* > 2σ(*I*)
Radiation source: fine-focus sealed tube	*R*_int_ = 0.059
φ and ω scans	θ_max_ = 25.3°, θ_min_ = 2.4°
Absorption correction: multi-scan (*SADABS*; Bruker, 2008)	*h* = −13→13
*T*_min_ = 0.835, *T*_max_ = 0.862	*k* = −15→15
44659 measured reflections	*l* = −16→16
6753 independent reflections	

### Refinement

**Table:**

Refinement on *F*^2^	Primary atom site location: structure-invariant direct methods
Least-squares matrix: full	Secondary atom site location: difference Fourier map
*R*[*F*^2^ > 2σ(*F*^2^)] = 0.036	Hydrogen site location: mixed
*wR*(*F*^2^) = 0.086	H-atom parameters constrained
*S* = 1.05	*w* = 1/[σ^2^(*F*_o_^2^) + (0.0358*P*)^2^ + 1.4431*P*] where *P* = (*F*_o_^2^ + 2*F*_c_^2^)/3
6753 reflections	(Δ/σ)_max_ = 0.001
523 parameters	Δρ_max_ = 0.26 e Å^−^^3^
0 restraints	Δρ_min_ = −0.41 e Å^−^^3^

### Special details

**Table:**

Geometry. All esds (except the esd in the dihedral angle between two l.s. planes) are estimated using the full covariance matrix. The cell esds are taken into account individually in the estimation of esds in distances, angles and torsion angles; correlations between esds in cell parameters are only used when they are defined by crystal symmetry. An approximate (isotropic) treatment of cell esds is used for estimating esds involving l.s. planes.

### Fractional atomic coordinates and isotropic or equivalent isotropic displacement parameters (Å^2^)

**Table:**

	*x*	*y*	*z*	*U*_iso_*/*U*_eq_	
Mn	0.88152 (3)	0.04367 (3)	0.20851 (2)	0.01081 (9)	
S	1.10820 (5)	0.19791 (5)	0.42751 (4)	0.02139 (14)	
F1	1.00980 (15)	0.40991 (12)	0.38570 (11)	0.0370 (4)	
F2	0.97858 (18)	0.33973 (14)	0.54012 (12)	0.0517 (5)	
F3	1.16153 (17)	0.36360 (14)	0.47867 (13)	0.0528 (5)	
N1	0.84244 (15)	−0.10052 (14)	0.22339 (12)	0.0122 (4)	
N2	0.70036 (15)	0.11097 (14)	0.25129 (13)	0.0127 (4)	
N3	0.91427 (15)	0.19154 (14)	0.17965 (13)	0.0126 (4)	
N4	1.04477 (15)	−0.01412 (14)	0.12917 (13)	0.0126 (4)	
O1	0.94653 (14)	0.00719 (12)	0.35015 (11)	0.0191 (3)	
H1O1	0.9612	0.0559	0.3758	0.029*	
H2O1	0.9150	−0.0320	0.3960	0.029*	
O2	1.16554 (17)	0.13077 (15)	0.51405 (13)	0.0330 (4)	
O3	1.19208 (15)	0.20862 (16)	0.33828 (13)	0.0368 (5)	
O4	0.98825 (15)	0.18025 (14)	0.41556 (13)	0.0263 (4)	
C1	0.92919 (19)	−0.19910 (17)	0.21380 (15)	0.0130 (4)	
C2	0.8723 (2)	−0.28720 (18)	0.25639 (16)	0.0160 (5)	
H2	0.9116	−0.3613	0.2617	0.019*	
C3	0.7507 (2)	−0.24308 (18)	0.28749 (16)	0.0159 (5)	
H3	0.6908	−0.2813	0.3169	0.019*	
C4	0.73116 (19)	−0.12714 (17)	0.26691 (15)	0.0129 (4)	
C5	0.61668 (19)	−0.05325 (17)	0.28771 (15)	0.0135 (4)	
C6	0.60387 (19)	0.05814 (17)	0.27983 (15)	0.0139 (4)	
C7	0.49042 (19)	0.13390 (18)	0.31028 (16)	0.0169 (5)	
H7	0.4117	0.1195	0.3294	0.020*	
C8	0.51858 (19)	0.23006 (18)	0.30619 (16)	0.0179 (5)	
H8	0.4634	0.2934	0.3238	0.021*	
C9	0.64944 (19)	0.21664 (17)	0.26963 (16)	0.0147 (4)	
C10	0.71524 (19)	0.29641 (17)	0.25885 (16)	0.0149 (5)	
C11	0.83947 (19)	0.28350 (17)	0.21575 (15)	0.0136 (4)	
C12	0.90644 (19)	0.36721 (17)	0.19642 (15)	0.0152 (5)	
H12	0.8766	0.4366	0.2131	0.018*	
C13	1.0209 (2)	0.32687 (17)	0.14944 (16)	0.0158 (5)	
H13	1.0843	0.3634	0.1282	0.019*	
C14	1.02682 (19)	0.21810 (17)	0.13828 (15)	0.0131 (4)	
C15	1.12767 (19)	0.15067 (17)	0.08940 (15)	0.0133 (4)	
C16	1.13167 (19)	0.04401 (17)	0.08165 (15)	0.0141 (4)	
C17	1.2333 (2)	−0.02581 (18)	0.03017 (16)	0.0177 (5)	
H17	1.3016	−0.0056	−0.0097	0.021*	
C18	1.2119 (2)	−0.12660 (18)	0.05006 (16)	0.0178 (5)	
H18	1.2634	−0.1885	0.0269	0.021*	
C19	1.09597 (19)	−0.12092 (17)	0.11317 (15)	0.0127 (4)	
C20	1.04806 (19)	−0.21024 (17)	0.15937 (15)	0.0133 (4)	
C21	0.50146 (19)	−0.09467 (17)	0.32576 (16)	0.0136 (4)	
C22	0.4326 (2)	−0.11748 (18)	0.26001 (17)	0.0175 (5)	
H22	0.4625	−0.1140	0.1929	0.021*	
C23	0.3192 (2)	−0.14545 (19)	0.29440 (18)	0.0229 (5)	
H23	0.2729	−0.1597	0.2501	0.027*	
C24	0.2747 (2)	−0.15223 (19)	0.39465 (19)	0.0252 (6)	
H24	0.1983	−0.1700	0.4173	0.030*	
C25	0.3445 (2)	−0.13248 (19)	0.46090 (18)	0.0249 (5)	
H25	0.3157	−0.1381	0.5283	0.030*	
C26	0.4576 (2)	−0.10425 (19)	0.42650 (17)	0.0220 (5)	
H26	0.5045	−0.0916	0.4712	0.026*	
C27	0.65225 (19)	0.39954 (17)	0.29927 (16)	0.0166 (5)	
C28	0.5486 (2)	0.47481 (17)	0.25887 (18)	0.0204 (5)	
H28	0.5179	0.4627	0.2042	0.024*	
C29	0.4914 (2)	0.56791 (19)	0.30047 (19)	0.0262 (6)	
H29	0.4219	0.6177	0.2737	0.031*	
C30	0.5363 (2)	0.58722 (19)	0.38062 (19)	0.0277 (6)	
H30	0.4972	0.6498	0.4080	0.033*	
C31	0.6399 (2)	0.5135 (2)	0.42083 (18)	0.0267 (6)	
H31	0.6708	0.5267	0.4749	0.032*	
C32	0.6974 (2)	0.41993 (19)	0.38034 (17)	0.0211 (5)	
H32	0.7668	0.3704	0.4076	0.025*	
C33	1.23923 (19)	0.19472 (17)	0.04546 (16)	0.0143 (4)	
C34	1.2315 (2)	0.27465 (18)	−0.03917 (16)	0.0186 (5)	
H34	1.1560	0.3021	−0.0677	0.022*	
C35	1.3358 (2)	0.31373 (19)	−0.08132 (16)	0.0207 (5)	
H35	1.3305	0.3661	−0.1388	0.025*	
C36	1.4477 (2)	0.27500 (18)	−0.03809 (17)	0.0201 (5)	
H36	1.5176	0.3014	−0.0663	0.024*	
C37	1.4554 (2)	0.19683 (19)	0.04725 (18)	0.0217 (5)	
H37	1.5302	0.1715	0.0769	0.026*	
C38	1.3521 (2)	0.15609 (18)	0.08875 (17)	0.0193 (5)	
H38	1.3581	0.1029	0.1456	0.023*	
C39	1.12663 (19)	−0.32285 (17)	0.15003 (16)	0.0144 (5)	
C40	1.1623 (2)	−0.35598 (18)	0.05741 (17)	0.0188 (5)	
H40	1.1365	−0.3076	0.0003	0.023*	
C41	1.2358 (2)	−0.46031 (19)	0.04959 (18)	0.0240 (5)	
H41	1.2608	−0.4808	−0.0128	0.029*	
C42	1.2722 (2)	−0.53402 (19)	0.13410 (19)	0.0240 (5)	
H42	1.3207	−0.6042	0.1289	0.029*	
C43	1.2360 (2)	−0.50259 (19)	0.22619 (19)	0.0251 (5)	
H43	1.2595	−0.5522	0.2832	0.030*	
C44	1.1650 (2)	−0.39772 (18)	0.23441 (17)	0.0200 (5)	
H44	1.1428	−0.3771	0.2968	0.024*	
C45	1.0622 (3)	0.3343 (2)	0.45958 (18)	0.0303 (6)	

### Atomic displacement parameters (Å^2^)

**Table:**

	*U*^11^	*U*^22^	*U*^33^	*U*^12^	*U*^13^	*U*^23^
Mn	0.00810 (16)	0.01012 (17)	0.01448 (17)	−0.00357 (12)	0.00103 (12)	−0.00258 (12)
S	0.0188 (3)	0.0274 (3)	0.0197 (3)	−0.0098 (2)	−0.0019 (2)	−0.0023 (2)
F1	0.0468 (9)	0.0266 (8)	0.0406 (9)	−0.0118 (7)	−0.0137 (7)	−0.0019 (7)
F2	0.0748 (12)	0.0514 (11)	0.0325 (9)	−0.0204 (9)	0.0149 (9)	−0.0241 (8)
F3	0.0723 (12)	0.0507 (11)	0.0550 (11)	−0.0442 (10)	−0.0334 (9)	0.0059 (9)
N1	0.0103 (9)	0.0125 (9)	0.0144 (9)	−0.0038 (7)	0.0003 (7)	−0.0032 (7)
N2	0.0103 (9)	0.0111 (9)	0.0177 (9)	−0.0049 (7)	−0.0001 (7)	−0.0024 (7)
N3	0.0108 (9)	0.0115 (9)	0.0159 (9)	−0.0034 (7)	0.0002 (7)	−0.0032 (7)
N4	0.0102 (8)	0.0137 (9)	0.0148 (9)	−0.0049 (7)	0.0010 (7)	−0.0037 (7)
O1	0.0246 (8)	0.0204 (8)	0.0165 (8)	−0.0124 (7)	−0.0038 (7)	−0.0019 (6)
O2	0.0372 (10)	0.0343 (11)	0.0300 (10)	−0.0187 (9)	−0.0115 (8)	0.0081 (8)
O3	0.0204 (9)	0.0542 (13)	0.0262 (10)	−0.0013 (8)	0.0057 (7)	−0.0016 (9)
O4	0.0204 (8)	0.0283 (10)	0.0358 (10)	−0.0113 (7)	−0.0036 (7)	−0.0106 (8)
C1	0.0138 (10)	0.0115 (11)	0.0148 (11)	−0.0031 (9)	−0.0032 (8)	−0.0037 (8)
C2	0.0162 (11)	0.0119 (11)	0.0203 (12)	−0.0037 (9)	−0.0017 (9)	−0.0030 (9)
C3	0.0144 (11)	0.0168 (11)	0.0186 (11)	−0.0088 (9)	−0.0001 (9)	−0.0024 (9)
C4	0.0123 (10)	0.0151 (11)	0.0134 (10)	−0.0070 (9)	0.0002 (8)	−0.0033 (8)
C5	0.0127 (10)	0.0170 (11)	0.0124 (10)	−0.0060 (9)	−0.0004 (8)	−0.0033 (8)
C6	0.0108 (10)	0.0162 (11)	0.0152 (11)	−0.0050 (9)	0.0000 (8)	−0.0029 (9)
C7	0.0089 (10)	0.0189 (12)	0.0226 (12)	−0.0044 (9)	−0.0001 (9)	−0.0030 (9)
C8	0.0106 (10)	0.0142 (11)	0.0255 (12)	−0.0008 (9)	0.0033 (9)	−0.0032 (9)
C9	0.0114 (10)	0.0127 (11)	0.0190 (11)	−0.0022 (9)	0.0001 (9)	−0.0030 (9)
C10	0.0136 (11)	0.0119 (11)	0.0180 (11)	−0.0022 (9)	−0.0013 (9)	−0.0013 (9)
C11	0.0141 (11)	0.0124 (11)	0.0142 (11)	−0.0034 (9)	−0.0013 (8)	−0.0020 (9)
C12	0.0171 (11)	0.0119 (11)	0.0167 (11)	−0.0042 (9)	−0.0002 (9)	−0.0029 (9)
C13	0.0144 (11)	0.0150 (11)	0.0192 (11)	−0.0078 (9)	0.0001 (9)	−0.0015 (9)
C14	0.0118 (10)	0.0136 (11)	0.0148 (11)	−0.0056 (8)	−0.0024 (8)	−0.0001 (9)
C15	0.0118 (10)	0.0162 (11)	0.0124 (10)	−0.0056 (9)	−0.0006 (8)	−0.0010 (9)
C16	0.0113 (10)	0.0169 (11)	0.0145 (11)	−0.0049 (9)	−0.0006 (8)	−0.0020 (9)
C17	0.0121 (10)	0.0207 (12)	0.0206 (12)	−0.0069 (9)	0.0045 (9)	−0.0050 (9)
C18	0.0148 (11)	0.0168 (12)	0.0218 (12)	−0.0029 (9)	0.0023 (9)	−0.0079 (9)
C19	0.0116 (10)	0.0143 (11)	0.0128 (10)	−0.0026 (8)	−0.0019 (8)	−0.0037 (8)
C20	0.0119 (10)	0.0143 (11)	0.0154 (11)	−0.0025 (9)	−0.0034 (8)	−0.0058 (9)
C21	0.0099 (10)	0.0099 (10)	0.0205 (11)	−0.0018 (8)	0.0002 (9)	−0.0035 (9)
C22	0.0169 (11)	0.0176 (12)	0.0186 (11)	−0.0058 (9)	−0.0036 (9)	−0.0006 (9)
C23	0.0166 (11)	0.0220 (13)	0.0329 (14)	−0.0087 (10)	−0.0104 (10)	0.0000 (10)
C24	0.0121 (11)	0.0203 (13)	0.0407 (15)	−0.0066 (10)	0.0020 (10)	0.0006 (11)
C25	0.0239 (13)	0.0257 (13)	0.0245 (13)	−0.0118 (11)	0.0111 (10)	−0.0054 (10)
C26	0.0236 (12)	0.0260 (13)	0.0203 (12)	−0.0119 (10)	0.0022 (10)	−0.0081 (10)
C27	0.0134 (11)	0.0126 (11)	0.0238 (12)	−0.0075 (9)	0.0083 (9)	−0.0049 (9)
C28	0.0148 (11)	0.0130 (11)	0.0312 (13)	−0.0057 (9)	0.0047 (10)	−0.0013 (10)
C29	0.0170 (12)	0.0147 (12)	0.0416 (15)	−0.0040 (10)	0.0086 (11)	−0.0015 (11)
C30	0.0265 (13)	0.0144 (12)	0.0402 (15)	−0.0101 (10)	0.0176 (11)	−0.0091 (11)
C31	0.0323 (14)	0.0251 (13)	0.0270 (13)	−0.0175 (11)	0.0115 (11)	−0.0108 (11)
C32	0.0187 (12)	0.0185 (12)	0.0257 (13)	−0.0078 (10)	0.0059 (10)	−0.0044 (10)
C33	0.0151 (11)	0.0119 (11)	0.0170 (11)	−0.0052 (9)	0.0035 (9)	−0.0064 (9)
C34	0.0163 (11)	0.0230 (12)	0.0176 (11)	−0.0075 (9)	−0.0007 (9)	−0.0033 (9)
C35	0.0241 (12)	0.0224 (12)	0.0162 (11)	−0.0113 (10)	0.0026 (9)	−0.0004 (9)
C36	0.0153 (11)	0.0223 (13)	0.0248 (12)	−0.0100 (10)	0.0080 (9)	−0.0092 (10)
C37	0.0103 (11)	0.0241 (13)	0.0307 (13)	−0.0046 (9)	0.0001 (10)	−0.0062 (10)
C38	0.0165 (11)	0.0149 (11)	0.0245 (12)	−0.0044 (9)	−0.0001 (9)	−0.0001 (9)
C39	0.0086 (10)	0.0136 (11)	0.0228 (12)	−0.0063 (9)	0.0002 (9)	−0.0040 (9)
C40	0.0199 (12)	0.0176 (12)	0.0201 (12)	−0.0049 (9)	−0.0045 (9)	−0.0035 (9)
C41	0.0223 (12)	0.0229 (13)	0.0301 (13)	−0.0052 (10)	0.0000 (10)	−0.0145 (11)
C42	0.0160 (11)	0.0137 (12)	0.0412 (15)	0.0000 (9)	−0.0016 (10)	−0.0079 (11)
C43	0.0180 (12)	0.0193 (13)	0.0320 (14)	−0.0008 (10)	−0.0023 (10)	0.0038 (11)
C44	0.0156 (11)	0.0209 (12)	0.0200 (12)	−0.0015 (9)	0.0017 (9)	−0.0022 (10)
C45	0.0404 (15)	0.0339 (15)	0.0239 (13)	−0.0219 (13)	−0.0076 (12)	−0.0013 (11)

### Geometric parameters (Å, º)

**Table:**

Mn—N1	1.9893 (17)	C17—H17	0.9300
Mn—N3	1.9912 (17)	C18—C19	1.431 (3)
Mn—N4	2.0044 (17)	C18—H18	0.9300
Mn—N2	2.0079 (17)	C19—C20	1.397 (3)
Mn—O1	2.1057 (15)	C20—C39	1.497 (3)
S—O3	1.4313 (17)	C21—C22	1.392 (3)
S—O2	1.4475 (18)	C21—C26	1.392 (3)
S—O4	1.4482 (16)	C22—C23	1.390 (3)
S—C45	1.820 (3)	C22—H22	0.9300
F1—C45	1.342 (3)	C23—C24	1.388 (3)
F2—C45	1.333 (3)	C23—H23	0.9300
F3—C45	1.336 (3)	C24—C25	1.385 (3)
N1—C4	1.386 (3)	C24—H24	0.9300
N1—C1	1.388 (3)	C25—C26	1.388 (3)
N2—C9	1.386 (3)	C25—H25	0.9300
N2—C6	1.388 (3)	C26—H26	0.9300
N3—C11	1.386 (3)	C27—C32	1.391 (3)
N3—C14	1.392 (3)	C27—C28	1.395 (3)
N4—C16	1.387 (3)	C28—C29	1.389 (3)
N4—C19	1.390 (3)	C28—H28	0.9300
O1—H1O1	0.8437	C29—C30	1.371 (4)
O1—H2O1	0.8225	C29—H29	0.9300
C1—C20	1.399 (3)	C30—C31	1.386 (4)
C1—C2	1.431 (3)	C30—H30	0.9300
C2—C3	1.352 (3)	C31—C32	1.386 (3)
C2—H2	0.9300	C31—H31	0.9300
C3—C4	1.428 (3)	C32—H32	0.9300
C3—H3	0.9300	C33—C34	1.390 (3)
C4—C5	1.394 (3)	C33—C38	1.393 (3)
C5—C6	1.390 (3)	C34—C35	1.387 (3)
C5—C21	1.499 (3)	C34—H34	0.9300
C6—C7	1.432 (3)	C35—C36	1.384 (3)
C7—C8	1.349 (3)	C35—H35	0.9300
C7—H7	0.9300	C36—C37	1.384 (3)
C8—C9	1.437 (3)	C36—H36	0.9300
C8—H8	0.9300	C37—C38	1.387 (3)
C9—C10	1.390 (3)	C37—H37	0.9300
C10—C11	1.394 (3)	C38—H38	0.9300
C10—C27	1.499 (3)	C39—C44	1.394 (3)
C11—C12	1.433 (3)	C39—C40	1.395 (3)
C12—C13	1.351 (3)	C40—C41	1.388 (3)
C12—H12	0.9300	C40—H40	0.9300
C13—C14	1.427 (3)	C41—C42	1.384 (3)
C13—H13	0.9300	C41—H41	0.9300
C14—C15	1.392 (3)	C42—C43	1.379 (3)
C15—C16	1.392 (3)	C42—H42	0.9300
C15—C33	1.495 (3)	C43—C44	1.386 (3)
C16—C17	1.431 (3)	C43—H43	0.9300
C17—C18	1.353 (3)	C44—H44	0.9300
			
N1—Mn—N3	174.02 (7)	N4—C19—C20	125.14 (18)
N1—Mn—N4	89.12 (7)	N4—C19—C18	109.24 (18)
N3—Mn—N4	89.97 (7)	C20—C19—C18	125.35 (19)
N1—Mn—N2	89.98 (7)	C19—C20—C1	122.82 (19)
N3—Mn—N2	89.29 (7)	C19—C20—C39	118.81 (18)
N4—Mn—N2	164.27 (7)	C1—C20—C39	118.38 (18)
N1—Mn—O1	92.84 (6)	C22—C21—C26	119.10 (19)
N3—Mn—O1	93.14 (7)	C22—C21—C5	120.39 (19)
N4—Mn—O1	98.57 (6)	C26—C21—C5	120.38 (19)
N2—Mn—O1	97.16 (7)	C23—C22—C21	120.1 (2)
O3—S—O2	115.56 (11)	C23—C22—H22	119.9
O3—S—O4	115.55 (11)	C21—C22—H22	119.9
O2—S—O4	114.10 (10)	C24—C23—C22	120.3 (2)
O3—S—C45	103.65 (12)	C24—C23—H23	119.8
O2—S—C45	103.18 (11)	C22—C23—H23	119.8
O4—S—C45	102.25 (11)	C25—C24—C23	119.8 (2)
C4—N1—C1	105.76 (16)	C25—C24—H24	120.1
C4—N1—Mn	126.66 (14)	C23—C24—H24	120.1
C1—N1—Mn	126.07 (13)	C24—C25—C26	119.8 (2)
C9—N2—C6	105.76 (16)	C24—C25—H25	120.1
C9—N2—Mn	126.80 (13)	C26—C25—H25	120.1
C6—N2—Mn	127.04 (14)	C25—C26—C21	120.7 (2)
C11—N3—C14	105.63 (16)	C25—C26—H26	119.6
C11—N3—Mn	126.19 (13)	C21—C26—H26	119.6
C14—N3—Mn	126.89 (14)	C32—C27—C28	119.1 (2)
C16—N4—C19	105.98 (16)	C32—C27—C10	119.0 (2)
C16—N4—Mn	126.78 (14)	C28—C27—C10	121.9 (2)
C19—N4—Mn	127.21 (13)	C29—C28—C27	119.8 (2)
Mn—O1—H1O1	121.1	C29—C28—H28	120.1
Mn—O1—H2O1	120.2	C27—C28—H28	120.1
H1O1—O1—H2O1	106.1	C30—C29—C28	120.7 (2)
N1—C1—C20	125.04 (18)	C30—C29—H29	119.7
N1—C1—C2	109.52 (17)	C28—C29—H29	119.7
C20—C1—C2	124.94 (19)	C29—C30—C31	120.0 (2)
C3—C2—C1	107.47 (19)	C29—C30—H30	120.0
C3—C2—H2	126.3	C31—C30—H30	120.0
C1—C2—H2	126.3	C30—C31—C32	119.9 (2)
C2—C3—C4	107.52 (19)	C30—C31—H31	120.1
C2—C3—H3	126.2	C32—C31—H31	120.1
C4—C3—H3	126.2	C31—C32—C27	120.5 (2)
N1—C4—C5	125.91 (19)	C31—C32—H32	119.7
N1—C4—C3	109.65 (18)	C27—C32—H32	119.7
C5—C4—C3	124.44 (19)	C34—C33—C38	119.23 (19)
C6—C5—C4	123.34 (19)	C34—C33—C15	120.34 (19)
C6—C5—C21	117.17 (18)	C38—C33—C15	120.43 (19)
C4—C5—C21	119.42 (18)	C35—C34—C33	120.4 (2)
N2—C6—C5	125.75 (19)	C35—C34—H34	119.8
N2—C6—C7	109.63 (18)	C33—C34—H34	119.8
C5—C6—C7	124.33 (19)	C36—C35—C34	120.2 (2)
C8—C7—C6	107.52 (18)	C36—C35—H35	119.9
C8—C7—H7	126.2	C34—C35—H35	119.9
C6—C7—H7	126.2	C37—C36—C35	119.8 (2)
C7—C8—C9	107.46 (19)	C37—C36—H36	120.1
C7—C8—H8	126.3	C35—C36—H36	120.1
C9—C8—H8	126.3	C36—C37—C38	120.3 (2)
N2—C9—C10	125.64 (18)	C36—C37—H37	119.8
N2—C9—C8	109.51 (18)	C38—C37—H37	119.8
C10—C9—C8	124.8 (2)	C37—C38—C33	120.1 (2)
C9—C10—C11	123.1 (2)	C37—C38—H38	119.9
C9—C10—C27	118.79 (18)	C33—C38—H38	119.9
C11—C10—C27	118.01 (18)	C44—C39—C40	118.3 (2)
N3—C11—C10	125.85 (19)	C44—C39—C20	120.49 (19)
N3—C11—C12	109.62 (17)	C40—C39—C20	121.18 (19)
C10—C11—C12	124.42 (19)	C41—C40—C39	120.7 (2)
C13—C12—C11	107.48 (19)	C41—C40—H40	119.7
C13—C12—H12	126.3	C39—C40—H40	119.7
C11—C12—H12	126.3	C42—C41—C40	120.3 (2)
C12—C13—C14	107.60 (18)	C42—C41—H41	119.8
C12—C13—H13	126.2	C40—C41—H41	119.8
C14—C13—H13	126.2	C43—C42—C41	119.5 (2)
N3—C14—C15	125.19 (19)	C43—C42—H42	120.3
N3—C14—C13	109.66 (18)	C41—C42—H42	120.3
C15—C14—C13	125.08 (19)	C42—C43—C44	120.5 (2)
C16—C15—C14	123.68 (19)	C42—C43—H43	119.7
C16—C15—C33	118.41 (18)	C44—C43—H43	119.7
C14—C15—C33	117.88 (18)	C43—C44—C39	120.7 (2)
N4—C16—C15	125.79 (19)	C43—C44—H44	119.7
N4—C16—C17	109.47 (18)	C39—C44—H44	119.7
C15—C16—C17	124.51 (19)	F2—C45—F3	107.6 (2)
C18—C17—C16	107.46 (18)	F2—C45—F1	107.3 (2)
C18—C17—H17	126.3	F3—C45—F1	107.1 (2)
C16—C17—H17	126.3	F2—C45—S	111.41 (17)
C17—C18—C19	107.71 (19)	F3—C45—S	111.2 (2)
C17—C18—H18	126.1	F1—C45—S	112.04 (17)
C19—C18—H18	126.1		
			
C4—N1—C1—C20	169.3 (2)	Mn—N4—C19—C20	−7.5 (3)
Mn—N1—C1—C20	−24.0 (3)	C16—N4—C19—C18	−3.5 (2)
C4—N1—C1—C2	−2.9 (2)	Mn—N4—C19—C18	178.36 (14)
Mn—N1—C1—C2	163.78 (14)	C17—C18—C19—N4	1.8 (2)
N1—C1—C2—C3	2.7 (2)	C17—C18—C19—C20	−172.4 (2)
C20—C1—C2—C3	−169.5 (2)	N4—C19—C20—C1	12.1 (3)
C1—C2—C3—C4	−1.4 (2)	C18—C19—C20—C1	−174.7 (2)
C1—N1—C4—C5	−177.8 (2)	N4—C19—C20—C39	−168.24 (19)
Mn—N1—C4—C5	15.7 (3)	C18—C19—C20—C39	5.0 (3)
C1—N1—C4—C3	2.1 (2)	N1—C1—C20—C19	4.3 (3)
Mn—N1—C4—C3	−164.52 (14)	C2—C1—C20—C19	175.3 (2)
C2—C3—C4—N1	−0.4 (2)	N1—C1—C20—C39	−175.41 (19)
C2—C3—C4—C5	179.4 (2)	C2—C1—C20—C39	−4.4 (3)
N1—C4—C5—C6	−9.4 (3)	C6—C5—C21—C22	101.0 (2)
C3—C4—C5—C6	170.8 (2)	C4—C5—C21—C22	−82.1 (3)
N1—C4—C5—C21	173.97 (19)	C6—C5—C21—C26	−74.9 (3)
C3—C4—C5—C21	−5.8 (3)	C4—C5—C21—C26	101.9 (2)
C9—N2—C6—C5	−170.4 (2)	C26—C21—C22—C23	2.4 (3)
Mn—N2—C6—C5	2.7 (3)	C5—C21—C22—C23	−173.6 (2)
C9—N2—C6—C7	3.6 (2)	C21—C22—C23—C24	−0.8 (3)
Mn—N2—C6—C7	176.68 (14)	C22—C23—C24—C25	−0.9 (4)
C4—C5—C6—N2	−0.3 (3)	C23—C24—C25—C26	1.1 (4)
C21—C5—C6—N2	176.46 (19)	C24—C25—C26—C21	0.5 (4)
C4—C5—C6—C7	−173.4 (2)	C22—C21—C26—C25	−2.2 (3)
C21—C5—C6—C7	3.3 (3)	C5—C21—C26—C25	173.8 (2)
N2—C6—C7—C8	−3.5 (3)	C9—C10—C27—C32	111.9 (2)
C5—C6—C7—C8	170.5 (2)	C11—C10—C27—C32	−65.5 (3)
C6—C7—C8—C9	2.0 (2)	C9—C10—C27—C28	−67.2 (3)
C6—N2—C9—C10	175.0 (2)	C11—C10—C27—C28	115.4 (2)
Mn—N2—C9—C10	1.9 (3)	C32—C27—C28—C29	−0.7 (3)
C6—N2—C9—C8	−2.3 (2)	C10—C27—C28—C29	178.37 (19)
Mn—N2—C9—C8	−175.45 (14)	C27—C28—C29—C30	0.5 (3)
C7—C8—C9—N2	0.2 (3)	C28—C29—C30—C31	0.1 (3)
C7—C8—C9—C10	−177.2 (2)	C29—C30—C31—C32	−0.5 (3)
N2—C9—C10—C11	8.3 (3)	C30—C31—C32—C27	0.2 (3)
C8—C9—C10—C11	−174.7 (2)	C28—C27—C32—C31	0.3 (3)
N2—C9—C10—C27	−168.9 (2)	C10—C27—C32—C31	−178.76 (19)
C8—C9—C10—C27	8.0 (3)	C16—C15—C33—C34	109.7 (2)
C14—N3—C11—C10	176.3 (2)	C14—C15—C33—C34	−72.0 (3)
Mn—N3—C11—C10	−16.0 (3)	C16—C15—C33—C38	−70.2 (3)
C14—N3—C11—C12	0.0 (2)	C14—C15—C33—C38	108.1 (2)
Mn—N3—C11—C12	167.73 (14)	C38—C33—C34—C35	1.4 (3)
C9—C10—C11—N3	−1.0 (3)	C15—C33—C34—C35	−178.5 (2)
C27—C10—C11—N3	176.29 (19)	C33—C34—C35—C36	−1.4 (3)
C9—C10—C11—C12	174.8 (2)	C34—C35—C36—C37	0.2 (3)
C27—C10—C11—C12	−7.9 (3)	C35—C36—C37—C38	0.9 (3)
N3—C11—C12—C13	−0.2 (2)	C36—C37—C38—C33	−0.8 (3)
C10—C11—C12—C13	−176.6 (2)	C34—C33—C38—C37	−0.3 (3)
C11—C12—C13—C14	0.3 (2)	C15—C33—C38—C37	179.6 (2)
C11—N3—C14—C15	−177.1 (2)	C19—C20—C39—C44	118.9 (2)
Mn—N3—C14—C15	15.3 (3)	C1—C20—C39—C44	−61.4 (3)
C11—N3—C14—C13	0.2 (2)	C19—C20—C39—C40	−61.7 (3)
Mn—N3—C14—C13	−167.42 (14)	C1—C20—C39—C40	118.0 (2)
C12—C13—C14—N3	−0.3 (2)	C44—C39—C40—C41	−1.0 (3)
C12—C13—C14—C15	177.0 (2)	C20—C39—C40—C41	179.56 (19)
N3—C14—C15—C16	−4.6 (3)	C39—C40—C41—C42	1.7 (3)
C13—C14—C15—C16	178.6 (2)	C40—C41—C42—C43	−0.8 (3)
N3—C14—C15—C33	177.27 (18)	C41—C42—C43—C44	−0.8 (3)
C13—C14—C15—C33	0.4 (3)	C42—C43—C44—C39	1.4 (3)
C19—N4—C16—C15	−170.7 (2)	C40—C39—C44—C43	−0.5 (3)
Mn—N4—C16—C15	7.4 (3)	C20—C39—C44—C43	178.9 (2)
C19—N4—C16—C17	4.0 (2)	O3—S—C45—F2	177.39 (18)
Mn—N4—C16—C17	−177.90 (14)	O2—S—C45—F2	−61.8 (2)
C14—C15—C16—N4	−7.3 (3)	O4—S—C45—F2	56.9 (2)
C33—C15—C16—N4	170.85 (19)	O3—S—C45—F3	−62.62 (19)
C14—C15—C16—C17	178.8 (2)	O2—S—C45—F3	58.23 (19)
C33—C15—C16—C17	−3.1 (3)	O4—S—C45—F3	176.92 (17)
N4—C16—C17—C18	−3.0 (2)	O3—S—C45—F1	57.18 (19)
C15—C16—C17—C18	171.8 (2)	O2—S—C45—F1	178.03 (17)
C16—C17—C18—C19	0.7 (2)	O4—S—C45—F1	−63.28 (19)
C16—N4—C19—C20	170.7 (2)		

### Hydrogen-bond geometry (Å, º)

*Cg*2, *Cg*3, *Cg*4, *Cg*7, *Cg*9 and *Cg*11 are the centroids of the N2/C6–C9, N3/C11–C14, N4/C16–C19, Mn/N2/C9–C11/N3, C21–C26 and C33–C38 rings, respectively.

**Table:**

*D*—H···*A*	*D*—H	H···*A*	*D*···*A*	*D*—H···*A*
O1—H1*O*1···O4	0.84	1.91	2.745 (2)	171
O1—H2*O*1···O2^i^	0.82	1.90	2.715 (2)	171
C7—H7···O3^ii^	0.93	2.39	3.170 (3)	141
C44—H44···F2^i^	0.93	2.50	3.397 (3)	162
C23—H23···*Cg*4^ii^	0.93	2.85	3.603 (3)	139
C25—H25···*Cg*2^iii^	0.93	2.89	3.650 (3)	139
C30—H30···*Cg*9^iv^	0.93	2.82	3.610 (3)	144
C37—H37···*Cg*2^v^	0.93	2.97	3.676 (3)	133
C40—H40···*Cg*3^vi^	0.93	2.62	3.449 (2)	148
C42—H42···*Cg*11^vii^	0.93	2.89	3.631 (3)	137

Symmetry codes: (i) −*x*+2, −*y*, −*z*+1; (ii) *x*−1, *y*, *z*; (iii) −*x*+1, −*y*, −*z*+1; (iv) *x*, *y*+1, *z*; (v) *x*+1, *y*, *z*; (vi) −*x*+2, −*y*, −*z*; (vii) *x*, *y*−1, *z*.
